# Factors Influencing Corrected Count Increment After Platelet Transfusion in Thrombocytopenic Patients

**DOI:** 10.7759/cureus.46161

**Published:** 2023-09-28

**Authors:** Dhananjay Prasad Sahu, Minal Wasnik, Pankaj K Kannauje

**Affiliations:** 1 Transfusion Medicine, All India Institute of Medical Sciences, Raipur, Raipur, IND; 2 Transfusion Medicine and Blood Bank, All India Institute of Medical Sciences, Raipur, Raipur, IND; 3 General Medicine, All India Institute of Medical Sciences, Raipur, Raipur, IND

**Keywords:** non-immunological factors, single donor platelet, random donor platelet, splenomegaly, refractoriness, immunological factors, thrombocytopenia, platelet transfusion, corrected count increment

## Abstract

Background

Platelet transfusion is a life-saving procedure for thrombocytopenic patients. Platelet transfusions can be either prophylactic or therapeutic. Prophylactic platelet transfusion reduces the risk of bleeding before any specific procedure, whereas therapeutic platelet transfusion helps to control active bleeding. Evaluation of the response to platelet transfusion by calculating the corrected count increment (CCI) is important to determine the success of platelet transfusion and to plan subsequent patient management.

Methods

We conducted a prospective observational study in patients who received at least one unit of platelet concentrate (random donor platelet (RDP) or single donor platelet (SDP)) admitted under or seen by General Medicine. Patients over 60 years, multiparous females, and chemotherapy patients were excluded. The patient's pre-transfusion and post-transfusion platelet counts at one hour and 24 hours were taken, and CCI was calculated.

Results

We studied 60 patients during the study period, out of which 35 were males (58.33%) and 25 were females (41.67%). The mean age was 36.2 years, and the age range was from 15 to 60 years. The majority of patients were O-Rh positive (41.67%), followed by B-Rh positive (40%), A-Rh positive (11.67%), and least by AB-Rh positive (5%) and only one patient with O-Rh negative (1.67%). The logistic regression analysis showed that sepsis (p=0.025), splenomegaly (p=0.004), COVID-19 (p=0.016), dengue (p=0.028), systemic lupus erythematosus (SLE) (p=0.045), immune thrombocytopenic purpura (ITP) (p=0.003) and fever (p=0.007) significantly contributed to unsuccessful CCI. However, acute leukemia (p=0.238), active bleeding (p=0.147), disseminated intravascular coagulopathy (DIC) (p=0.952), aplastic anemia (AA) (p=0.114), and sickle cell disease (SCD) (p=0.739) did not show any statistical significance.

Conclusion

Unsuccessful CCI at 24 hours is attributed to non-immunological clinical factors like sepsis, splenomegaly, COVID-19, and fever, whereas immunological clinical factors like SLE, dengue, and ITP resulted in unsuccessful CCI at one hour, as evident by this study.

## Introduction

Platelet transfusion is considered a supportive and effective therapy for patients with thrombocytopenia. It is used for the prevention or treatment of bleeding in cases of low platelet count or poor platelet function. The decision regarding platelet transfusion depends on the clinical condition of the patient, the cause of thrombocytopenia, the platelet count, and the functional ability of the patient‘s own platelets [1,2]. Patients receive either single donor platelets (SDP) prepared by apheresis or random donor platelets (RDP) prepared from whole blood donations.

A standard dose of platelets (six units of whole blood-derived RDP or one SDP) generally increases the platelet count by about 30000-40000  platelets/μL/unit in a 70-kg adult [3]. The efficacy of platelet transfusion is assessed by the corrected count increment (CCI). We undertook this study to assess various clinical factors responsible for the failure of the corrected count increment at one hour and 24 hours.

## Materials and methods

After getting Institutional Ethics Committee approval (AIIMSRPR/IEC/2020/673), we performed this prospective observational study in the Department of Transfusion Medicine and Blood Bank of All India Institute of Medical Sciences, Raipur, India. The patients admitted to or seen by the Department of General Medicine were studied. We studied 60 patients for the duration of one year in 2021. The sample size was determined based on the patient load during the COVID-19 pandemic.

The inclusion criteria were: patients who gave consent; ≥ 15 years and ≤ 60 years; both genders; and patients who received one standard dose (either 6 units of RDP or one unit of SDP) of platelet transfusion. However, exclusion criteria were: patients who refused to take part in the study; patients having a failure of the platelet products to be completely transfused; multiparous females (more than three children); repeated admission of the same patient; patients who have qualitative defects like Bernard Soulier syndrome (BSS); Glanzmann thrombasthenia (GT); and patients with malignancy on chemotherapy.

We received a requisition for platelet transfusions from the Department of General Medicine for thrombocytopenic patients. The patient’s demographic and other basic characteristics like age, gender, blood group, height, weight, parity in females, and diagnosis were noted. The relevant clinical factors like fever, bleeding, splenomegaly, sepsis, disseminated intravascular coagulation (DIC), etc. were also noted. The patient’s platelet count was also noted before the transfusion of platelet units (random donor platelet (RDP) or single donor platelet (SDP)). The platelet units were prepared as per the standard operating procedures (SOP) of the department. As per Indian national standards for blood centers and blood transfusion services by the National Blood Transfusion Council, for a unit of 350 ml or 450 ml of whole blood-derived RDP, the minimum threshold for platelet count is 3.5x1010 and 4.5x1010 per bag, respectively. Whereas for SDP, the minimum threshold for platelet count is 3x1011 per bag [4]. The platelet count of each RDP or SDP was determined before issuing. The efficacy of platelet transfusion is assessed by CCI. The patient’s post-transfusion platelet count at one hour and 24 hours was determined. All the counts were performed on a calibrated Sysmex XP-100 three-part automated cell counter. The pre-transfusion and post-transfusion platelet counts were determined, and the difference between them was the platelet count increment or absolute platelet increment. The CCI following platelet transfusion at one hour and 24 hours was calculated as below:

Patient’s body surface area (BSA) was calculated by Mosteller’s formula as [5]:

$$BSA (m2)= height (cm) x weight (kg) / 3600$$

\(CCI = Absolute platelet Increment/\mu L x Body surface area (m^{2})/ Number of platelet transfused (10^{11})\)

[6].

The IBM SPSS Statistics for Windows, Version 21.0 (Released 2012; IBM Corp., Armonk, New York, United States) was used to statistically evaluate the data, p-value <0.05 was considered significant.

## Results

We studied 60 patients during the study period, out of which 35 were males (58.33%) and 25 were females (41.67%). The majority of patients were 15-30 years old (n=24, 40%), whereas the number of patients with ages in the range of 30-45 years and 45-60 years was 17 and 19, respectively. The mean age was 36.2 years, and the age range was from 15-60 years. The mean height was 158.3 cm (range 127-177.8 cm), and the mean weight was 55.1 kg (range 30-83 kg). The mean body surface area of the patients was 1.55 m2 (range 1.02-1.96 m2). The majority of patients were O-Rh positive, seen in 25 (41.67%), followed by B-Rh positive in 24 (40%), A-Rh positive in 7 (11.67%), and least by AB-Rh positive in 3 (5%), and only one patient with O-Rh negative (1.67%). The patients receiving platelet transfusions had the various diagnoses shown in Table 1. These patients received platelet transfusions due to associated thrombocytopenia. The most common clinical condition was COVID-19, followed by newly diagnosed acute leukemias. Other diagnoses included immune thrombocytopenic purpura (ITP), aplastic anemia, dengue, DIC, etc.

**Table 1:** Summary table of 60 patients as per diagnosis of the patients SLE: systematic lupus erythematosus; ITP: immune thrombocytopenic purpura; DIC: disseminated intravascular coagulation

S. No.	Diagnosis	Number of cases	Percentage of cases(%)
1.	Systematic lupus erythematosus (SLE)	4	6.67
2.	Acute leukemia	9	15
3.	COVID-19	10	16.67
4.	Immune thrombocytopenic purpura (ITP)	8	13.33
5.	Aplastic anemia	7	11.67
6.	Sickle cell disease	6	10
7.	Dengue	3	5
8.	Disseminated intravascular coagulation (DIC)	1	1.67
9.	Hemorrhagic shock	1	1.67
10.	Chronic kidney disease	4	6.67
11.	Cerebrovascular disease	2	3.30
12.	Tuberculosis	1	1.67
13.	Testicular malignancy	1	1.67
14.	Malaria	1	1.67
15.	Acute hepatitis	1	1.67
16.	Plasma cell disorder	1	1.67
	Total	60	100

The effect of various clinical factors on these 60 patients was studied by a logistic regression analysis (Table 2). The analysis showed that the presence of sepsis, splenomegaly, COVID-19, dengue, systemic lupus erythematosus (SLE), ITP, and fever significantly contributed to unsuccessful platelet increment, whereas acute leukemia, active bleeding, DIC, aplastic anemia, and sickle cell disease (SCD) did not show any statistically significant effect on platelet increment.

**Table 2:** Logistic regression analysis of various clinical factors BMI: body mass index; DIC: disseminated intravascular coagulopathy; ITP: immune thrombocytopenic purpura; SLE: systemic lupus erythematosus; CCI: corrected count increment; SCD: sickle cell disease

Clinical factors	95% confidence interval		p-value
Non-immunological factors	Lower	Upper	
Fever	0.092	0.546	0.007
Sepsis	-0.540	-0.039	0.025
Splenomegaly	-0.684	-0.138	0.004
BMI >25	-0.454	-0.454	0.283
Acute leukemia	-0.399	0.102	0.238
Active bleeding	-0.374	0.058	0.147
COVID-19	-0.698	-0.077	0.016
DIC	-0.549	0.583	0.952
Aplastic anemia	-0.210	0.953	0.114
SCD	-0.284	0.398	0.739
Immunological factors			
ITP	-0.778	-0.168	0.003
SLE	-0.830	-0.010	0.045
Dengue	-0.972	-0.057	0.028

**Figure 1:**
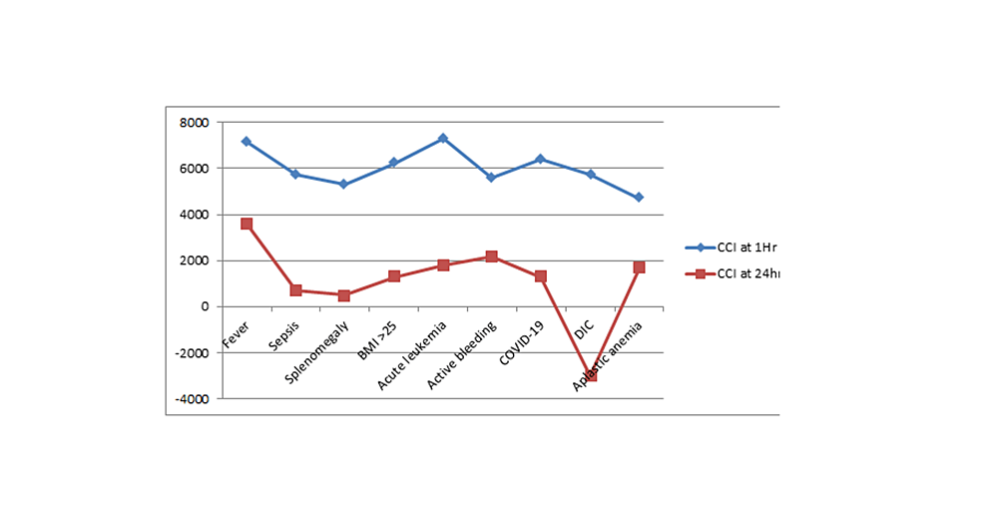
The influence of non-immunological factors on one hour and 24 hours post-transfusion platelet increment and CCI X-axis indicates non-immunological factors and Y-axis indicates CCI CCI: corrected count increment; DIC: disseminated intravascular coagulation; BMI: body mass index

**Figure 2:**
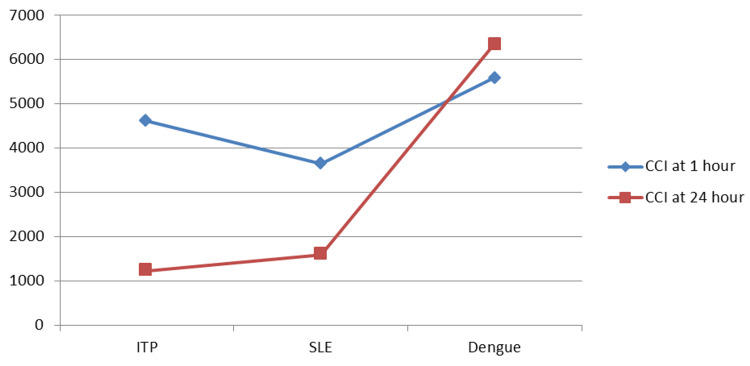
The influence of immunological factors on one hour and 24 hours post-transfusion platelet increment and CCI X-axis indicates non-immunological factors and Y-axis indicates CCI CCI: corrected count increment; ITP: immune thrombocytopenic purpura; SLE: systemic lupus erythematosus

It was observed that non-immunological factors such as fever, sepsis, splenomegaly, BMI > 25, acute leukemia, active bleeding, COVID-19, DIC, and aplastic anemia have a decreasing effect on CCI at 24 hours as compared to that at one hour, as shown in Figure 1. Immunological factors have resulted in a decrease in CCI at 1 hour and 24 hours, except for dengue (a smaller sample size), as shown in Figure 2.

In Table 3, many clinical parameters impacting the response to platelet transfusion are analyzed. The logistic regression analysis shows that the presence of sepsis (p=0.025), splenomegaly (p=0.004), COVID-19 (p=0.016), dengue (p=0.028), SLE (p=0.045), ITP (p=0.003), and fever (p=0.007) significantly contributed to unsuccessful CCI. But acute leukemia (p=0.238), active bleeding (p=0.147), DIC (p=0.952), aplastic anemia (AA) (p=0.114), and SCD (p=0.739) do not show any statistical significance. A p value < 0.05 is considered statistically significant.

**Table 3:** The influence of various clinical factors on one hour and 24 hours absolute platelet increment and CCI BMI: body mass index; DIC: disseminated intravascular coagulopathy; ITP: immune thrombocytopenic purpura; SLE: systemic lupus erythematosus; CCI: corrected count increment

Variable	Number of factors	One hour after the transfusion		24 hours after transfusion	
Non-immunological factors		Absolute platelet increment (Platelet count/microliter)	CCI	Absolute platelet increment ( Platelet count/microliter)	CCI
Fever	40	15200	7137.85	8075	3593
Sepsis	16	10500	5732.99	1500	711.45
Splenomegaly	12	12250	5292.6	1916.66	507.08
BMI >25	9	9777	6228.72	2222	1291.87
Acute leukemia	12	17416	7314.83	3583	1777.61
Active bleeding	22	12818	5603.65	5463.63	2163.51
COVID-19	10	12600	6405.68	3200	1291.84
DIC	2	11500	5742.39	-3500	-2992.37
Aplastic anemia	8	7750	4697.39	2625	1660.65
Immunological factors					
ITP	10	10200	4617.27	2720	1231.33
SLE	5	7000	3654.54	2600	1599.4
Dengue	4	12000	5588.69	13750	6362.86

## Discussion

CCI is a parameter to measure the response to platelet transfusion in patients. In the present study, we studied 60 patients who received SDP or RDP, and CCI of platelets was observed at one hour and 24 hours of transfusion. CCI at one hour was observed to be successful for 22 (36.67%) patients out of 60 patients and further declined to only 11 (18.33%) patients with successful CCI at 24 hours. Unsuccessful CCI at one hour was found for 38 patients, out of which CCI for four patients increased, and for the rest, 34 patients remained unsuccessful at 24 hours. Among 45 patients who showed unsuccessful CCI at 24 hours, there were 11 patients who, despite one hour of successful CCI, failed to have the desired increment at 24 hours. As per Hod E et al., platelet refractoriness is the term given to a consistent failure to achieve an appropriate platelet count increment following platelet transfusion. There were various non-immune factors like fever, sepsis, splenomegaly, DIC, bleeding, etc., and immune factors like human leukocyte antigens (HLA) and human platelet antigens (HPA) antibodies and ABO incompatibility involved in it [7].

In our study, we performed the logistic regression analysis for various clinical factors, which showed that sepsis, splenomegaly, fever, COVID-19, dengue, SLE, and ITP significantly contributed to unsuccessful CCI. Sepsis was observed in 16 patients out of the total 60 patients studied. A study by Shastry et al. showed a decreasing effect of sepsis on platelet count [8], which could be due to the fact that our patients included mainly medicine patients, with the majority of them being in the intensive care unit (ICU). In our study, we also found that patients with splenomegaly had unsuccessful CCI, which was statistically significant. In splenomegaly, there is platelet pooling in the enlarged spleen, and various studies have also shown a significant decrease in platelet count [8,9,10,11]. Fever was present in 40 out of 60 patients, of whom 70% had unsuccessful CCI. Various studies have observed a significant decrease in platelet count in line with our study [9,10,12,13], and some studies have also shown a significant influence of fever, such as Kumawat et al. [14]. The reason quoted by several authors for the cause of unsuccessful CCI in fever could be due to the promotion of endothelial cell activation by elevated cytokines IL-1 and TNF-α. It could also be attributed to the fact that all these patients were also associated with other clinical factors, namely COVID-19, sepsis, etc. Thrombocytopenia in COVID-19 patients can be attributed to platelet apoptosis, the incorporation of platelets into microthrombi (peripheral consumption), and severe thrombotic events [15].

In ITP and SLE, there is antibody formation against the platelets, leading to immune-mediated platelet destruction [16]. We found a significant influence of these on platelet count increments. Prawita et al., in their study on 35 patients, observed that four patients had ITP, out of which three (75%) showed unsuccessful CCI [16]. In our study, we had eight patients with ITP and four patients with SLE, all of whom had unsuccessful CCI.

We studied the various clinical factors in thrombocytopenic patients in medical settings, which could be considered a limitation of this study. Although we found certain clinical factors to be statistically significant, we recommend further studies including a larger patient population with various clinical illnesses in different clinical settings to fully understand the significance of different underlying clinical factors.

## Conclusions

We found that certain non-immunological clinical factors like sepsis, splenomegaly, COVID-19, and fever showed unsuccessful CCI at 24 hours, which was statistically significant, whereas immunological clinical factors like dengue, SLE, and ITP were found to be statistically significant causes of unsuccessful CCI at one hour. Identifying these underlying causes and treating them would help in getting a better response to platelet transfusion in patients requiring platelet transfusion.

## References

[1] Lieberman L, Bercovitz RS, Sholapur NS, Heddle NM, Stanworth SJ, Arnold DM (2014). Platelet transfusions for critically ill patients with thrombocytopenia. Blood.

[2] Nahirniak S, Slichter SJ, Tanael S (2015). Guidance on platelet transfusion for patients with hypoproliferative thrombocytopenia. Transfus Med Rev.

[3] Simon TL, McCullough J, Snyder EL, Solheim BG, Strauss RG (2016). Rossi's Principles of Transfusion Medicine, 5th Edition. https://www.wiley.com/en-us/Rossi%27s+Principles+of+Transfusion+Medicine%2C+5th+Edition-p-9781119013013.

[4] (2023). National Standards for Blood Centres and Blood Transfusion Services (2nd Edition). https://main.mohfw.gov.in/newshighlights-99.

[5] Mosteller RD (1987). Simplified calculation of body-surface area. N Engl J Med.

[6] Matsui R, Hagino T, Tsuno NH (2021). Does time of CCI measurement affect the evaluation of platelet transfusion effectiveness?. Transfus Apher Sci.

[7] Hod E, Schwartz J (2008). Platelet transfusion refractoriness. Br J Haematol.

[8] Shastry S, Chaudhary R (2012). Clinical factors influencing corrected count increment. Transfus Apher Sci.

[9] Slichter SJ, Davis K, Enright H (2005). Factors affecting posttransfusion platelet increments, platelet refractoriness, and platelet transfusion intervals in thrombocytopenic patients. Blood.

[10] Senthil E (2020). Evaluation of Corrected Count Increment in Hemato-Oncological Patients Receiving Single Donor Platelets Transfusion. http://repository-tnmgrmu.ac.in/13900/.

[11] Bishop JF, McGrath K, Wolf MM (1988). Clinical factors influencing the efficacy of pooled platelet transfusions. Blood.

[12] Bishop JF, Matthews JP, McGrath K, Yuen K, Wolf MM, Szer J (1991). Factors influencing 20-hour increments after platelet transfusion. Transfusion.

[13] Alcorta I, Pereira A, Ordinas A (1996). Clinical and laboratory factors associated with platelet transfusion refractoriness: a case-control study. Br J Haematol.

[14] Kumawat V, Goyal M, Marimuthu P (2020). Analysis of donor safety in high yield plateletpheresis procedures: an experience from tertiary care hospital in South India. Indian J Hematol Blood Transfus.

[15] Rohlfing AK, Rath D, Geisler T, Gawaz M (2021). Platelets and COVID-19. Hamostaseologie.

[16] Prawita AAAL, Mulyantari NK, Herawati S (2019). The description of corrected count increment on one hour and 24 hours. after platelet apheresis transfusion in Sanglah General Hospital Denpasar. Bali Med J.

